# Functional Abdominal Pain and Nutritional Status of Children. A School-Based Study

**DOI:** 10.3390/nu12092559

**Published:** 2020-08-24

**Authors:** Amanda C. Fifi, Carlos Velasco-Benitez, Miguel Saps

**Affiliations:** 1 Department of Pediatric Gastroenterology, Hepatology and Nutrition, University of Miami/Miller School of Medicine, Miami, FL 33136, USA; msaps@med.miami.edu; 2 Department of Pediatrics, Universidad del Valle, Cali 760001, Colombia; carlos.velasco@correounivalle.edu.co

**Keywords:** functional abdominal pain, obesity, irritable bowel syndrome, functional dyspepsia, functional gastrointestinal disorders

## Abstract

Functional abdominal pain disorders (FAPD) are the most common chronic pain conditions in pediatric gastroenterology. They account for 50% of all pediatric gastroenterology clinic visits. The pathophysiology of FAPDs is poorly understood, but there is growing understanding of the role of food and the patient’s nutritional state in both their treatment and prognosis. Clinic-based studies have shown a higher prevalence of FAPDs, and worse prognosis among obese children with FAPDs. We aimed to assess the nutritional status of children with FAPD to determine if there is increased prevalence of FAPDs in obese or underweight patients. We conducted a cross sectional study of schoolchildren in Colombia. We enrolled 1030 patients from five schools and screened them for FAPDs using Rome IV criteria. Data on weight, height, abdominal circumference and BMI were collected for each child. Cases (FAPDs) were compared with a control group of enrolled children who did not meet diagnostic criteria for any functional gastrointestinal disorders (FGID). We diagnosed 58 (5.8%) children with FAPDs based on Rome IV criteria. When we compared to participants who were not diagnosed with FGIDs by screening, there was no statistically significant difference in children who were obese (OR 0.34 CI: 0.03–1.34, *p* = 0.124) or overweight (OR 1.00 CI: 0.46–2.02, *p* = 0.984) or those with increased abdominal circumference (OR 0.94, CI: 0.10–3.90, *p* = 0.943). FAPDs are not more common among obese children compared with healthy controls at a community level. Obese children may have been overrepresented in previous studies which were done at a clinical level due to comorbidities and a more severe phenotype that makes them more likely to consult. Nutritional status is not a useful predictor for the occurrence of FAPDs in children in the general population.

## 1. Introduction

Functional abdominal pain disorders (FAPD) are among the most common types of chronic pain disorders in children [1]. FAPDs account for 2% to 4% of all general pediatric office visits [2] and more than 50% of all consultations to pediatric gastroenterology [3]. The last edition of the Rome criteria (Rome IV) subdivide FAPDs in four distinct diagnoses: irritable bowel syndrome (IBS), functional dyspepsia (FD), functional abdominal pain—not otherwise specified (FAP—NOS) and abdominal migraine [4].

The pathophysiology of FAPDs remains poorly understood. Multiple factors seem to be involved. Among those factors, food and nutritional status are thought to play a role in triggering symptoms and influencing their prognosis [5]. Multiple studies performed in clinic settings have found an association between malnutrition and FAPDs [6,7]. However, the relation between the specific type of malnutrition and FAPDs is not clear. Both obesity and being underweight have been considered risk factors for different types of FAPDs in different studies. A Polish study using Rome III criteria to diagnose FAPDs, found a higher frequency of abnormal growth in children with FAPDs compared to healthy children [6]. The results were somewhat conflicting and difficult to explain as excessive body weight was most commonly found in children with IBS while children with functional abdominal pain syndrome were more likely to be underweight. Conversely, an American study that also used the Rome III classification to diagnose children at a general pediatric clinic, found an association between both, functional abdominal pain syndrome and IBS with being obese/overweight [8]. Additionally, a recent Israeli retrospective study of pediatric gastroenterology clinic patients diagnosed with FAPDs per the Rome IV diagnostic criteria, found that adolescents with FAPDs had a significantly higher prevalence of overweight/obesity compared to a population data base control group [7]. An important limitation of these studies is their potential for selection bias, as all of them were conducted in patients who presented for consultation. As a result, these studies are less likely to include patients with less severe symptoms, poor access to care or those who visit the doctor less frequently, such as adolescents. Thus, the published studies may not reflect the true relationship between FAPDs and nutritional status in the general populace. In addition, some of the studies, included control groups from population-based data sets and not from within the same patient population that was screened for FAPDs.

We conducted a community-based cross-sectional study to assess the relationship between nutritional status and FAPDs in schoolchildren in Colombia. The study has the potential to advance our understanding of the “true” relationship between FAPDs and nutritional state by overcoming some of the limitations of previous studies.

## 2. Methods

We sent invitation letters to the homes of all children aged 10–18 years from 5 schools in Cali, Colombia. Participants were screened for FAPDs using Rome IV criteria. Children who met criteria for FD, IBS, FAP—NOS and abdominal migraine were considered cases and those without any functional gastrointestinal disorder (FGID) served as controls. Patients with organic gastrointestinal disease or disease affecting growth were excluded from the study.

A member of the research team performed anthropometric measurements on all participants including weight, height and abdominal circumference measurements. Body height (centimeters) was measured from the soles of the participant’s feet to the top of head. Body weight (kilograms) was measured with an electronic scale. Abdominal circumference (centimeters) was measured at level of the umbilicus. Body mass index (BMI) was calculated as weight in kilograms divided by the square of the height in meters.

Anthropometric measurements were adjusted for age and sex. Children were classified as overweight when BMI was between +1 and +2 standard deviations and obese when BMI was above +2 standard deviations according to sex-specific World Health Organization (WHO) growth chart. Children were designated as underweight when the BMI was between −2 and −3 standard deviations and severe underweight when BMI was below −3 standard deviations according to the WHO growth chart. Abdominal obesity was defined as abdominal circumference >90% for age and sex on WHO growth chart.

### 2.1. Statistical Analysis

Data are presented as mean or percentages when appropriate. Anthropometric measurements are provided as age- and sex-specific. Standard deviations were calculated. To describe the distribution of variables, exploratory analysis was performed for all the variables. For the continuous variables, we obtained the central tendency and dispersion measurements. For categorical variables, we obtained frequencies and proportions. We estimated the proportion of children with its corresponding 95% confidence interval (CI); and the descriptive measurements with their corresponding standard deviations and ranges. To evaluate risk factors, a univariate analysis was initially performed between each of the exposure variables of interest and the effect variable. Odds ratios (OR) were calculated and 95% CI was included. Fisher’s exact test was used to compare the proportions of normal/abnormal nutritional state between the groups of children with and without FAPDs. *p*-values < 0.05 were considered significant.

### 2.2. Ethical Considerations

All study procedures were approved by the Institutional Review Board of the University of Valle (HUV 024-2019), and parental informed consent was obtained for all participants.

## 3. Results

### 3.1. Study Sample

We invited 1297 school children to participate. Eighty percent (1152/1297) accepted our invitation. One hundred and twenty-two (9.7%) children were excluded due to pre-existing medical conditions as outlined above. Thus, 1030 schoolchildren ages 10–18 years old (mean 13.9 (+/−1.9) years) completed the study, 531 (51.6%) girls and 499 (48.4%) boys (Table 1).

### 3.2. Nutritional Status

Seven hundred and thirteen children (69.2%) were normal weight. Ninety-five children (9.2%) were obese, 198 (19.2%) were overweight, 19 (1.8%) were underweight and 5 (0.5%) children were severely malnourished. Thirty-seven (3.7%) children were diagnosed with abdominal obesity (38.9% of all obese children).

### 3.3. Functional Abdominal Pain Disorders

We diagnosed 58 (5.8%) children with FAPDs based on Rome IV criteria, Table 1. Thirty-six children (3.6%) met criteria for FD (29 (2.9%) epigastric pain syndrome and 7 (0.7%) post-prandial distress syndrome). Twelve (1.2%) children had IBS (3 (0.3%) constipation type, 6 (0.6%) mixed type and 3 (0.3%) unspecified type). Six (0.6%) children were diagnosed with abdominal migraine and 4 (0.4%) with FAP—NOS. Children diagnosed with an FAPD were more likely to be girls, (OR 3.87, CI: 1.83–6.95, *p* = 0.000) and least likely to be white race (OR 0.25 CI: 0.04–0.79, *p* = 0.013) than the control group.

### 3.4. Relationship between Nutritional Status and FAPDs

When we compared the 58 children diagnosed with FAPDs by Rome IV criteria to participants who were not diagnosed with a FGID, we found no statistically significant difference in terms of nutritional state: obesity (OR 0.34 CI: 0.03–1.34, *p* = 0.124), overweight (OR 1.00 CI: 0.46–2.02, *p* = 0.984), abdominal obesity (OR 0.94, CI: 0.10–3.90, *p* = 0.943) (Table 2). We also did not find a significant difference in the subanalysis of the different FAPD diagnoses (FD, IBS, FAP—NOS and abdominal migraine) for nutritional state than healthy school children. We did not analyze the relation between being underweight and FAPDs because there were no underweight children diagnosed with FAPDs in our cohort.

## 4. Discussion

This is the first study to compare the nutritional status of children with FAPDs with healthy controls at a community level. Our study found no significant difference in the nutritional status between schoolchildren diagnosed with FAPD by Rome IV criteria and healthy controls in the community. This study reinforces the Rome IV criteria by showing that FAPDs are rarely associated with “red flag” symptoms such as weight loss, underweight or short stature.

While food is thought to play a role in triggering FAPDs [9], it is unclear whether anthropometrics influence the prevalence of FAPDs in the population at large, unlike other gastrointestinal diseases [10]. Though many foods have been implicated in exacerbation of gastrointestinal symptoms in pediatrics [5], the degree to which this affects growth is not well demonstrated. Dairy products, gluten and fermentable oligo-, di-, monosaccharides and polyols (FODMAPs) are commonly reported culprits [9]. Interestingly, some of these food groups are also frequently reported as source of intolerance in healthy controls [9]. However, even if some of these food groups could play a role in the development of signs/symptoms in a subgroup of children with FAPDs, the ingested amount reported to cause signs or symptoms is small and unlikely to affect the child’s weight or height. In fact, most children with disaccharidase deficiency are of normal weight [11].

Our findings seem to contradict the results of previous studies based at a medical clinic level that found an increased prevalence of FAPDs in obese children [7]. However, this contradiction may only be apparent as the design of previous studies made them prone to selection bias by only including children seeking medical care. Children who attend clinics are more likely to present symptoms, including abdominal pain, the most common cause of consultation in pediatric gastroenterology [3]. Obese children also seek medical services more often due to comorbidities, like depression, gastroesophageal reflux disease and other chronic pain disorders, [12] and as such, are more likely to be diagnosed with FAPDs than their normal weight counterparts. Moreover, children with FAPDs that are obese have a worse prognosis [13] making them more likely to consult and to be captured at the time of conducting a research study at the medical office. Similarly, underweight children are more likely to be referred for evaluation [14] and thus have an increased likelihood of being diagnosed with FAPDs after negative workup. There also may be a selection bias in office-based studies that include adolescents, as consultations for general health checkup are less frequent in this age group [15]. Previous studies that compared patients with FAPDs to population-based control groups may have also underdiagnosed FAPDs in the controls, as parents frequently underestimate their children’s abdominal pain and therefore are less likely to report those symptoms in general population surveys [16]. Our study overcame some of this problems by individually screening each child using Rome IV criteria to define cases and controls.

Nevertheless, we cannot ignore the results of previous studies that found obese patients presenting to clinics were more likely to have FAPDs [7], and that obese patients had more prolonged, severe symptoms [13]. Together, the results of the current and previous studies seem to suggest that although obesity is not associated with higher rates of FAPDs at the community level, it may be an identifier for a more aggressive phenotype of FAPDs in children. The confirmation of this assumption in future studies is of great importance as the identification of populations at risks could prevent worsened morbidity, associated poor quality of life and prognosis [17].

The strengths of our study include its large sample size and design that allowed us to assess the true difference between the diseased group and controls. Questionnaires were given at the school level to facilitate completion and reduce selection bias. Members of the research team conducted all measures to enhance the accuracy of results.

Our study is not without limitations. First, the results of this study may not be generalizable to other populations. We only measured weight, height and abdominal girth and calculated the BMI. BMI is widely used as an indicator of nutritional status [18]; however, obesity would be more accurately demonstrated using measurement of abdominal fat mass with dual-energy radiograph absorptiometry [19], which would be difficult to conduct in such a large number of children. In addition, there were no underweight children in the cohort of children diagnosed with FAPDs, which made comparisons impossible for this measure of nutritional status.

## 5. Conclusions

Our study suggests that the prevalence of FAPDs is similar in obese children and healthy controls at a community level. Nutritional status is not a useful predictor for the occurrence of FAPD in children in the general population. Obese children may be overrepresented in previous studies conducted at the clinical level due to higher rate of comorbidities and a more severe phenotype. Future research should confirm our findings and investigate the role of obesity in the development of more severe FAPDs and the use of weight control measures to mitigate these effects.

## Figures and Tables

**Table 1:** Demographics of study participants and cases of FAPDs.

	All Participants *n* = 1030	Cases of FAPDs *n* = 58
Mean Age/years	13.9 +/− 1.9	14.8 +/− 2.0
Age range/years	10–18	10–18
**Age groups**		
Elementary school	263 (25.5)	10 (17.2)
Adolescent	767 (74.5)	48 (82.8)
**Sex**		
Female	531 (51.6)	44 (75.9)
Male	499 (48.4)	14 (24.1)
**Race**		
“Mixed”	390 (58.3)	43 (74.1)
White	176 (26.3)	7 (12.1)
African	79 (11.8)	7 (12.1)
Indigenous	24 (3.6)	1 (1.7)
**Weight according to WHO**		
Normal	713 (69.2)	45 (77.6)
Overweight/obese	293 (28.4)	13 (22.5)
Obese	95 (9.2)	2 (3.5)
Overweight	198 (19.2)	11 (19.0)
Underweight	24 (2.4)	0 (0.0)
Moderate underweight	19 (1.8)	0 (0.0)
Severe underweight	5 (0.5)	0 (0.0)
**Height according to WHO**		
Normal	991 (96.2)	56 (96.5)
Short stature	32 (3.1)	2 (3.5)
Moderate short stature	31 (3.0)	2 (3.5)
Severe short stature	1 (0.1)	0 (0.0)
Tall	7 (0.7)	0 (0.0)
**Abdominal circumference according to WHO**		
Normal	993 (96.4)	56 (96.5)
Abdominal obesity	37 (3.6)	2 (3.5)

FAPDs—functional abdominal pain disorders; *n*—number; WHO—World Health Organization.

**Table 2:** Children diagnosed with functional abdominal pain disorder by Rome IV criteria compared to control group of children without a functional gastrointestinal disorder.

	OR	CI 95%	*p*
**Groups**			
Elementary school age	1.00		
Adolescent	1.64	0.80–3.70	0.1594
**Sex**			
Male	1.00		
Female	3.47	1.83–6.95	0.0000
**Race**			
“Mixed”	1.00		
White	0.25	0.04–0.79	0.0134
African	0.68	0.13–2.21	0.5311
Indigenous	0.64	0.01–4.12	0.6649
**Weight according to WHO**			
Normal	1.00		
Malnutrition	0.64	0.31–1.24	0.1716
Obese/overweight	0.73	0.35–1.41	0.3344
Obese	0.34	0.03–1.34	0.1245
Overweight	1.00	0.46–2.02	0.9847
Underweight	n/a		
Severe underweight	n/a		
Moderate underweight	n/a		
**Height according to WHO**			
Normal	1.00		
Abnormal height	n/a		
Short stature	1.19	0.13–5.02	0.8118
Moderate short stature	1.19	0.13–5.02	0.8118
Severe short stature	n/a		
Tall	n/a		
**Abdominal circumference according to WHO**			
Normal	1.00		
Abdominal obesity	0.94	0.10–3.90	0.9436

OR—odds ratio; CI—confidence interval; WHO—World Health Organization.

## Abbreviations

FAPD	Functional abdominal pain disorder
IBS	Irritable bowel syndrome
FD	Functional dyspepsia
FAP—NOS	Functional abdominal pain—not otherwise specified
FGID	Functional gastrointestinal disorder
BMI	Body mass index
OR	Odds ratio
WHO	World Health Organization
CI	Confidence interval
FODMAPs	Fermentable oligo-, di-, monosaccharides and polyols
